# Evolution of rarity and phylogeny determine above- and belowground biomass in plant-plant interactions

**DOI:** 10.1371/journal.pone.0294839

**Published:** 2024-05-20

**Authors:** Alivia G. Nytko, Ashlynn M. Hord, John K. Senior, Julianne O’Reilly-Wapstra, Jennifer A. Schweitzer, Joseph K. Bailey

**Affiliations:** 1 Ecology and Evolutionary Biology, University of Tennessee, Knoxville, TN, United States of America; 2 Discipline of Biological Sciences, School of Natural Sciences, University of Tasmania, Tasmania, Australia; KGUT: Graduate University of Advanced Technology, ISLAMIC REPUBLIC OF IRAN

## Abstract

Rare species are often considered inferior competitors due to occupancy of small ranges, specific habitats, and small local populations. However, the phylogenetic relatedness and rarity level (level 1–7 and common) of interacting species in plant-plant interactions are not often considered when predicting the response of rare plants in a biotic context. We used a common garden of 25 species of Tasmanian *Eucalyptus*, to differentiate non-additive patterns in the biomass of rare versus common species when grown in mixtures varying in phylogenetic relatedness and rarity. We demonstrate that rare species maintain progressively positive non-additive responses in biomass when interacting with phylogenetically intermediate, less rare and common species. This trend is not reflected in common species that out-performed in monocultures compared to mixtures. These results offer predictability as to how rare species’ productivity will respond within various plant-plant interactions. However, species-specific interactions, such as those involving *E*. *globulus*, yielded a 97% increase in biomass compared to other species-specific interaction outcomes. These results are important because they suggest that plant rarity may also be shaped by biotic interactions, in addition to the known environmental and population factors normally used to describe rarity. Rare species may utilize potentially facilitative interactions with phylogenetically intermediate and common species to escape the effects of limiting similarity. Biotically mediated increases in rare plant biomass may have subsequent effects on the competitive ability and geographic occurrence of rare species, allowing rare species to persist at low abundance across plant communities. Through the consideration of species rarity and evolutionary history, we can more accurately predict plant-plant interaction dynamics to preserve unique ecosystem functions and fundamentally challenge what it means to be “rare”.

## Introduction

Abiotic and biotic factors jointly shape plant fitness by imposing a variety of selective pressures on performance traits. Plant performance traits, such as vegetative growth, reproduction, and survival reflect overarching patterns in plant fitness [1]. These traits are impacted by individual functional traits, such as specific leaf area, photosynthetic rate, and others [1]. Performance traits not only determine individual fitness, but also scale up to influence numerous facets of community composition (e.g., coexistence with other plant species, above- and belowground mutualisms), range dynamics, and ecosystem function (e.g., via the modulation of functional diversity within communities) [2,3]. Biomass is a key performance trait for plants [1] which is commonly thought to be shaped primarily by abiotic factors. However, biotic factors such as plant-plant interactions also influence plant growth, resource use, and responses to environmental change [4–6].

Facilitation (i.e., species relationships characterized by one or more species positively impacting the fitness of another species [7,8]) and competition (i.e., species relationships characterized by negative effects on species fitness caused by the presence of neighboring species, often caused by limiting resources [6]) are two of the most common outcomes of plant-plant interactions. Facilitation and competition can shift plant growth and dispersal traits, resulting in various outcomes including local adaptation, niche partitioning, and competitive exclusion [6]. For example, Brooker et al. [9] suggested that plant-plant facilitation can act as an evolutionary force, driving the selection of dispersal traits with long term impacts on niche expansion, contraction, convergence, and divergence. Furthermore, Beltrán et al. [10] found that patterns of facilitation and competition between congeneric species were also affected by the trait divergence of the interacting species. Consequently, congeners with large phenotypic differences in traits may experience less niche overlap, and therefore have increased facilitative interactions [10]. These outcomes alter local and global population dynamics, and therefore have the potential to alter species ranges by promoting or inhibiting range expansion and/or altering the range boundary shape [9,11–13]. Given the far-reaching impacts of plant-plant interactions across scales of biological organization, understanding the eco-evolutionary factors driving the outcomes of these interactions on plant performance (i.e., facilitation, competition, neutral) is critically important in an era of climate change and increasing anthropogenic disturbance.

**Fig 1:**
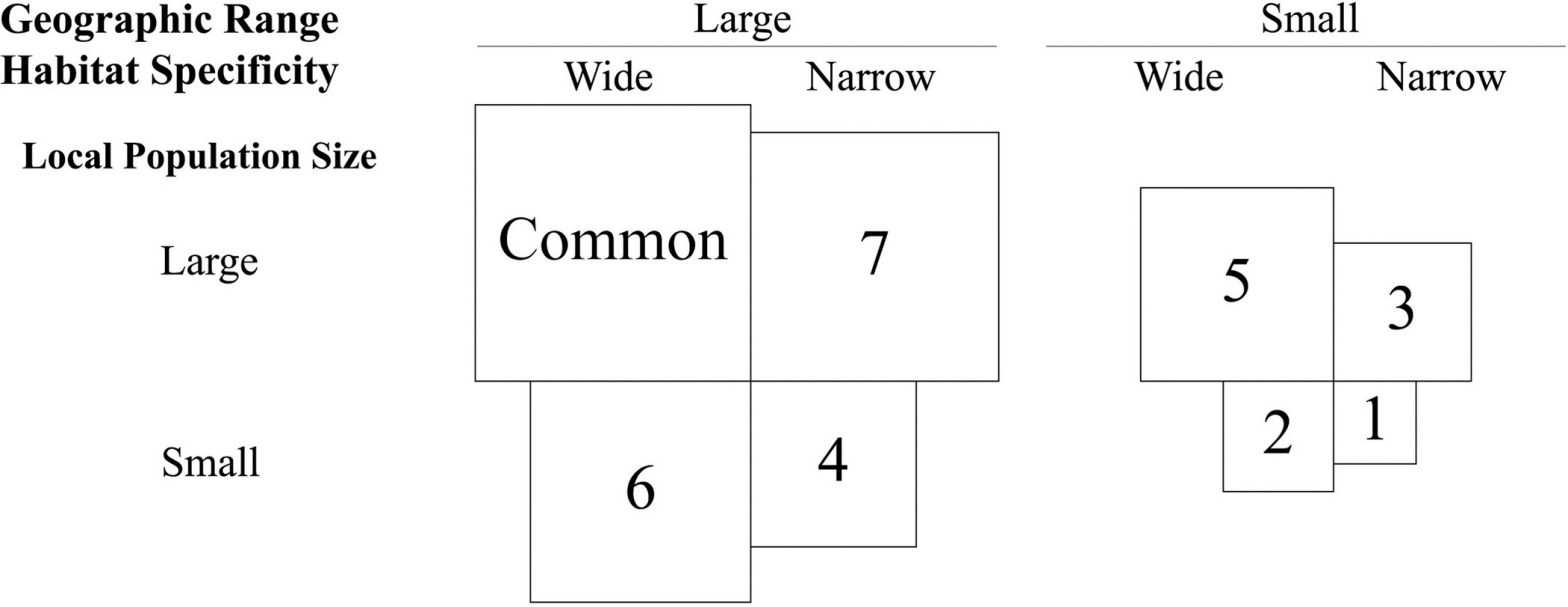
Classification of rank ordered rarity levels using geographic range, habitat specificity, and local population size [14]. Box size provides an abstract representation of geographic occurrence ranging from a highly specialized rarity level 1 to common. Twenty-five species of Tasmanian *Eucalyptus* were categorized into rarity levels based on range size, habitat specificity (ratio of bioregions inhabited in Tasmania, Australia), and local population aggregation (representative of local population size) [19].

Species rarity, which is commonly defined solely in terms of geographic occurrence (i.e., rare species have constrained ranges, high habitat specificity, and small local populations [14,15]), is an increasingly common phenomenon driven by a complex combination of ecological and evolutionary factors that shift geographic patterns of occurrence and persistence. Variation in the rarity of interacting species can change the strength and/or direction of biotic interactions within communities across a wide variety of taxonomic groups [16–18]. Therefore, accounting for species rarity in studies of plant-plant interactions will allow for a more nuanced and realistic understanding of how biotic interactions influence plant performance. To accomplish this, rarity “levels”, ranging from the most to least rare and common, are often assigned to species using an ordinally ranked system which accounts for each of the three aspects of geographic occurrence used to define rarity (**Table 1 and Fig 1**) [14]. This classification system provides a useful scaffold for investigating how rare species differ from each other as well as more common ones. It can thus be leveraged to better understand how factors influencing plant performance, such as biotic interactions, vary depending upon the rarity level of plants in a community.

**Table 1:** Classification of 25 species of Tasmanian eucalypts by genetic lineage (GL) [20,21], section, series, and rarity. Measures of range size, habitat specificity, and population aggregation from Williams & Potts [19] were used to group species into seven different ordinal rarity levels (Fig 1). Species range sizes were created by connecting occupied 10 km x 10 km grid cells from the outer marginal extremes of a core distribution to outliers and interpolating the number of cells within the resulting envelope for each species. Similarly, the aggregation of each species was calculated as the average percent occurrence within each grid cell of the species’ range. Additionally, habitat specificity was calculated for each species as the proportion of bioregions inhabited in Tasmania.

**GL 1**					
**Species**	**Section/Subsection**	**Range Size**	**Habitat Specificity**	**Aggregation**	**Rarity**
*E*. *risdonii*	Aromatica, Insulanae	4	1/9	100	1
*E*. *radiata*	Aromatica, Radiatae	7	2/9	88	1
*E*. *sieberi*	Cineracea, Psathyroxla	37	3/9	73	3
*E*. *pulchella*	Aromatica, Insulanae	126	5/9	70	3
*E*. *tenuiramis*	Aromatica, Insulanae	118	2/9	78	5
*E*. *nitida*	Aromatica, Insluanae	292	7/9	76	6
*E*. *regnans*	Eucalyptus, Regnantes	171	7/9	40	7
*E*. *obliqua*	Eucalyptus, Eucalyptus	436	9/9	68	Common
*E*. *delegatensis*	Cineracea, Fraxinales	349	9/9	68	Common
*E*. *amygdalina*	Aromatica, Insulanae	417	9/9	87	Common
**GL 2**					
**Species**	**Section/Series**	**Range Size**	**Habitat Specificity**	**Aggregation**	**Rarity**
*E*. *barberi*	Maidenaria, Foveolatae	17	1/9	68	3
*E*. *brookeriana*	Maidenaria, Foveolatae	81	6/9	22	4
*E*. *rodwayi*	Maidenaria, Foveolatae	135	7/9	36	6
*E*. *ovata*	Maidenaria, Foveolatae	412	9/9	49	Common
**GL 3**					
**Species**	**Section/Series**	**Range Size**	**Habitat Specificity**	**Aggregation**	**Rarity**
*E*. *urnigera*	Maidenaria, Orbiculares	29	3/9	21	2
*E*. *cordata*	Maidenaria, Orbiculares	25	2/9	37	3
*E*. *johnstonii*	Maidenaria, Semiunicolores	47	3/9	41	3
*E*. *subcrenulata*	Maidenaria, Semiunicolores	65	4/9	43	4
*E*. *vernicosa*	Maidenaria, Semiunicolores	76	4/9	41	5
*E*. *gunnii*	Maidenaria, Orbiculares	100	5/9	36	6
**GL 4**					
**Species**	**Section/Series**	**Range Size**	**Habitat Specificity**	**Aggregation**	**Rarity**
*E*. *perriniana*	Maidenaria, Orbiculares	4	1/9	14	1
*E*. *rubida*	Maidenaria, Viminales	86	5/9	55	4
*E*. *dalrympleana*	Maidenaria, Viminales	176	6/9	54	6
*E*. *globulus*	Maidenaria, Globulares	211	7/9	54	7
*E*. *viminalis*	Maidenaria, Viminales	424	9/9	71	Common

In addition to species rarity, the evolutionary history and phylogenetic context of plant species plays a large role in driving community assemblage and productivity [22,23]. For example, Perea et al. [23] demonstrated that less abundant saplings tend to be surrounded by more phylogenetically dissimilar or less closely related species, compared to common species in high abundance [23]. Similarly, Malecore et al. [24] found that introduced seedling establishment and growth was optimized within phylogenetically intermediate communities. Coexistence theory provides a mechanism to explain the outcomes of phylogenetically-based interactions through the examination of the interaction between the equalizing mechanisms (interspecific fitness differences) and stabilizing mechanisms (niche differentiation) that determine community composition and stability. One such explanation is limiting similarity, in which phylogenetically similar species tend to have similar trait values and niche requirements, leading to stronger competition for the same resources [25]. Additionally, the relative competitive ability and frequency of interacting species can impact plant persistence when niche differences lead to increased fitness for certain plant species when they exist at low density in the community (frequency-dependent regulation). Consequently, related species with divergent phenotypic traits can also experience competitive exclusion [25]. Therefore, critically examining the response of plant species varying in rarity level and phylogenetic relatedness to plant-plant interactions allows for more powerful inference of future patterns of competition, facilitation, community composition, and associated ecosystem function in rare species, especially as rarity intensifies under the influence of climate change [16].

To understand the eco-evolutionary dynamics underlying plant-plant interactions among rare and common species in a phylogenetic framework, we used a full factorial common garden experiment with 25 species of Tasmanian eucalypts of known phylogenetic relatedness and varying in rarity [26]. Previous work [26] showed a phylogenetic basis to performance traits associated with the major geographic determinants of rarity in these eucalypt species. However, little is known about how these trait values shift in response to plant-plant interactions varying in rarity and relatedness. We, therefore, hypothesized: 1) Total, aboveground, and belowground plant biomass will differ in two-species mixtures versus monoculture, as well as between degrees of phylogenetic relatedness within mixtures; 2) Total, aboveground, and belowground plant biomass of rare species will increase when interacting with progressively less rare neighboring species. Our results show that rare species have enhanced competitive abilities and synergistic non-additive responses in genetically intermediate relationships as well as in interactions with common plant species. These findings demonstrate the high potential for leveraging specific plant-plant interactions to increase the productivity and performance of rare plant species and allow for the maintenance of functionally unique ecosystems [27].

## Methods

### Common garden

Our common garden was comprised of 25 species of Tasmanian *Eucalyptus* which represented two subgenera (Symphyomyrtus and Eucalyptus), four phylogenetic sections (Maidenaria, Aromatica, Cineraceae, Eucalyptus), and five series (Globulares, Orbiculares, Viminales, Seminunicolores, Foveolatae) (see Wooliver et al. [20] for resolved phylogenetic relatedness). Each of the 25 species of native Tasmanian *Eucalyptus* was categorized into one of the seven ordinally ranked levels of rarity based on range size, habitat specificity, and population size in accordance with Rabinowitz [14] (Table 1). Species range sizes in Tasmania were derived from the methodology outlined by Williams & Potts [19]. This involved connecting occupied 10 km x 10 km grid cells from the outer marginal extremes of a core distribution to outliers and interpolating the number of cells within the resulting envelope for each species. Similarly, the aggregation of each species was calculated as the average percent occurrence within each grid cell of the species’ range. For instance, a species that has a range size of six 10 km x 10 km grid cells and only occurs within 20% of those cells has a range of 600 km^2^ and an aggregation of 20%. Additionally, we calculated habitat specificity for each species as the proportion of bioregions inhabited in Tasmania. The rarest species (level 1) demonstrated small range and population sizes across a limited number of bioregions, while common species demonstrated large range and population sizes across many bioregions in Tasmania. Each form of rarity was represented by at least one species of Tasmanian *Eucalyptus* (**S1 Fig**). A full factorial common garden experiment consisting of monocultures and mixtures of different species under varying levels of CO_2_ and Nitrogen (N) fertilization was developed using seeds from each species obtained from one to six maternal trees within a single population (**S2 Fig**). Each *Eucalyptus* species was grown in a two species pairing consisting of monocultures, as well as phylogenetically similar, intermediate, and distant congeners. Within the context of this study, phylogenetically similar pairings were comprised of species within the same subgenera, section, and series, and had a relative, continuous phylogenetic dissimilarity of 25% or lower; phylogenetically intermediate pairings were comprised of species within the same subgenera, but different sections or series, and had a relative, continuous phylogenetic dissimilarity of 25–50%; phylogenetically dissimilar pairings were comprised of species within different subgenera, and had a relative, continuous phylogenetic dissimilarity of 50% or greater. Monoculture treatments represented pairs of conspecific individuals. Interactions varying in phylogenetic relatedness spanned all rarity levels, such that each mixture type consisted of all possible rarity level combinations (S2 Fig). Rarity levels were assigned to both the target species (i.e. the randomized species of interest) and interacting species (i.e. the species planted in mixture with the target species) within pairings; therefore, they shall be referred to as target rarity level and interacting rarity level respectively. Continuous phylogenetic distances between species pairings were calculated across the Tasmanian *Eucalyptus* phylogeny provided in Wooliver et al. [20] (“cophenetic.phylo” function in “ape” package, R).

After approximately five months of growth, target species seedlings were harvested, and performance traits were measured. Specifically, above- and belowground biomass was separated, dried, and weighed (g). Above- and belowground biomass was summed to determine total biomass. Total seedling biomass is significantly positively correlated with mean adult height at maturity in Tasmanian eucalypts (r: 0.28, p-value = 1.602e-13) (**S3 Fig**). Although total seedling biomass is reflective of adult height in Tasmanian eucalypts, we recognize that there is a temporal component to plant community stability and composition [23] that we cannot directly capture through seedling measurements. See details of this experiment from Senior et al. [21]). Data from this paper were recategorized through the addition of rarity levels and reanalyzed using continuous phylogenetic distances to address the hypotheses outlined above. Recategorized data is available at Nytko [28].

### Statistical analyses

All statistical analyses were performed using R Statistical Software (version 4.2.1, R Core Team 2022). Step-wise model selection was performed on linear mixed models (LMM) with main and interactive effects of continuous phylogenetic relatedness, categorical interacting rarity level, CO_2_ addition, N fertilizer addition, and mean adult height on total biomass, aboveground biomass, and belowground biomass respectively (“lmer”, “AIC”, and “step” functions in “lme4” and “stats” packages, R). The models with the lowest Akaike information criterion (AIC) value were selected. The final models included the main effects of categorical interacting rarity level, continuous phylogenetic distance, CO_2_ addition, and N fertilizer enrichment, as well as interactive effects of interacting rarity x phylogenetic distance, phylogenetic distance x N fertilizer enrichment, and N fertilizer enrichment x CO_2_ addition, and random effect of target species identity, on total biomass, aboveground biomass, and belowground biomass separately. Interactions between CO_2_ addition, N fertilizer, and other effects remained in all models; however, the main effect of CO_2_ addition and N fertilizer enrichment on the biomass of eucalypts is detailed in Senior et al. [21].

To address both hypothesis 1 and 2, we examined the main and interactive effects of continuous phylogenetic distance and categorical interacting rarity, as well as the main effect of mean adult height, on the total, aboveground, and belowground biomass of target species, accounting for species-level differences as random variables in the model error structure. Species-specific effects on total, aboveground, and belowground biomass were accounted for through the creation of separate intercepts for each target species in all analyses. Although the effect of interacting rarity level and phylogenetic distance of neighboring species was examined separately for total, aboveground, and belowground biomass, total biomass was used to determine the interaction strength of two-species mixtures as to provide a holistic (total biomass = above + belowground biomass) view of overall seedling productivity in mixture versus monoculture. Specifically, to determine the strength of interactions between each pair of rarity levels, we first calculated the differences in observed mean biomass and expected mean biomass (based on total biomass production in monocultures). Then, we standardized each interaction strength by dividing each target species mean, by species-specific standard deviations ((observed mean total biomass—expected mean total biomass) / species standard deviation). To determine whether there were significant non-additive effects among species mixtures across all levels of target rarity, standardized interaction strengths were used in linear mixed models and one-sample T tests (mu = 0). Specifically, a linear mixed model was performed across all rarity levels to determine the main and interacting effects of continuous phylogenetic distance, categorical interacting rarity level, and mean adult height on the standard interaction strength of target species total biomass. Model selection was conducted by comparing the linear mixed model to a null model without predictors and utilizing step-wise model selection. Step-wise model selection recommended a linear mixed model examining the main and interacting effects of continuous phylogenetic distance, categorical interacting rarity level, and mean adult height, excluding species-level effects as a random variable. The AIC value of the selected LMM was 1366.65 compared to the null model AIC value of 1377.42. T-tests were used among each rarity level to separately determine if the average standardized interaction strength significantly differed from the expected null of 0, which corresponds with neutral plant-plant interactions. Positive interaction strengths in community mixtures represent synergistic non-additive effects indicative of facilitation, negative interaction strengths represent antagonistic non-additive effects indicative of competition, and neutral interaction strengths represent additive effects indicative of neutral plant-plant interactions [29].

## Results

**Fig 2:**
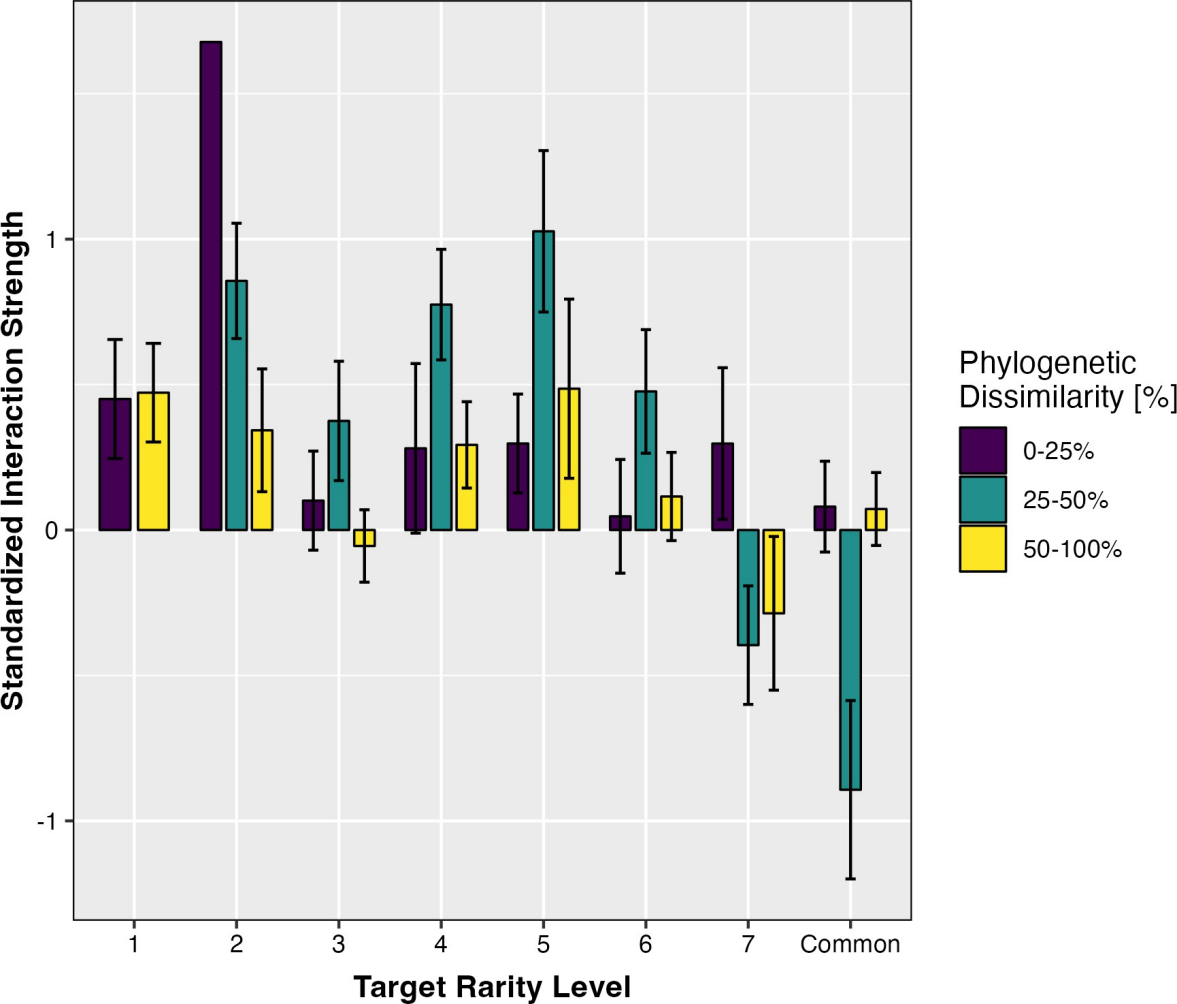
Comparative boxplots demonstrating the standardized interaction strengths of target species in pairings varying in target rarity level and phylogenetic dissimilarity. Positive standardized interaction strengths represent synergistic non-additivity in total biomass (species-specific pairing outperformed biomass expectation in respective monocultures) and negative standardized interaction strengths represent antagonistic non-additivity in total biomass (species-specific pairing underperformed biomass expectation in respective monocultures). Phylogenetic pairings that are 0–25% related are phylogenetically similar, 25–50% related are phylogenetically intermediate, and 50–100% related are phylogenetically dissimilar.

In support of hypothesis 1, the phylogenetic distance and rarity level of interacting neighbor species were strong, interacting, determinants of total and aboveground biomass in Tasmanian Eucalyptus (Fig 2 and Table 2). While the phylogenetic distance underlying plant-plant interactions did not significantly affect the belowground biomass of eucalypts, the rarity levels of interacting species, as well as the interaction between interacting rarity and phylogenetic distance, significantly influenced belowground biomass (Table 2).

**Table 2:** Results of linear mixed models, examining the main and interactive effects of categorical interacting rarity level (1–7 and common), continuous phylogenetic distance, CO_2_ addition, and N fertilizer enrichment on total, aboveground, and belowground biomass. Species-level effects were counted for in the model error structure using separate intercepts for each target species identification. Step-wise model selection was used to optimize the fit of each model. Alpha = 0.05.

Response	Effects	DF	Chisq	P value
Total Biomass	Interacting Rarity	7	42.997	3.341e-07*
	Phylogenetic Distance	1	4.263	0.039*
	N Fertilizer Enrichment	1	30.014	4.289e-08*
	CO_2_ Addition	1	31.210	2.316e-08*
	Interacting Rarity x Phylogenetic Distance	7	15.919	0.026*
	Phylogenetic Distance x N Fertilizer Enrichment	1	5.865	0.015*
	N Fertilizer Enrichment x CO_2_ Addition	1	26.951	2.086e-07*
Aboveground Biomass	Interacting Rarity	7	44.745	1.533e-07^*^
	Phylogenetic Distance	1	4.287	0.038*
	N Fertilizer Enrichment	1	36.397	1.609e-09*
	CO_2_ Addition	1	32.202	1.389e08*
	Interacting Rarity x Phylogenetic Distance	7	15.195	0.034*
	Phylogenetic Distance x N Fertilizer Enrichment	1	5.661	0.017*
	N Fertilizer Enrichment x CO_2_ Addition	1	31.764	1.741e-08*
Belowground Biomass	Interacting Rarity	7	33.999	1.723e-05*
	Phylogenetic Distance	1	3.238	0.072
	N Fertilizer Enrichment	1	9.238	0.002*
	CO_2_ Addition	1	21.523	3.497e-06*
	Interacting Rarity x Phylogenetic Distance	7	15.792	0.027*
	Phylogenetic Distance x N Fertilizer Enrichment	1	5.160	0.023*
	N Fertilizer Enrichment x CO_2_ Addition	1	9.781	0.002*

We found that rare eucalypts, except for the rarest species (i.e., level 1) had increased total, aboveground, and belowground biomass when interacting with phylogenetically intermediate partners (Fig 2 and Table 2). Phylogenetically similar and dissimilar pairings also increased the total, aboveground, and belowground biomass of rare species; however, not to the level of intermediately related pairings (Fig 2). For example, when paired with intermediately related species, moderately rare species belonging to levels 4, 5, and 6 had 84%, 117%, and 49% greater standardized interaction strengths than within phylogenetically similar pairings, and 109%, 80%, and 110% greater standardized interaction strengths than with phylogenetically distant pairings, respectively. The total biomass of the rarest species did not vary largely based on the phylogenetic distance of neighboring species, but rather displayed increased total, aboveground, and belowground biomass and positive standardized interaction strengths under all pairings compared to monocultures (Fig 2 and **Table 3**). While both phylogenetically similar and dissimilar pairings increased the biomass of the rarest species, the average total biomass only differed by 0.001g between phylogenetically similar and dissimilar pairings. In contrast, common species and those belonging to rarity level 7 had higher total biomass than all other species, but demonstrated increased antagonistic non-additivity when interacting with phylogenetically intermediate neighbors and neutral, additive responses overall (Fig 2 and Table 3). In other words, more common species demonstrated a unique ability to maintain high biomass in monocultures, but an inability to increase or retain high biomass in plant-plant interactions varying in phylogenetic relatedness. Although common species on average demonstrated 75% greater total biomass than species belonging to rarity level 2 in monocultures, the same rare species on average demonstrated 28% greater total biomass than common species when interacting with phylogenetically intermediate neighbors. Additionally, the average adult height of each target species did not significantly affect the biomass of eucalypts in pairs across all rarity levels and phylogenetic relationships.

**Table 3:** a) Results of one-sample t-tests (mu = 0) examining the difference between observed biomass in mixtures and the expected biomass based on biomass production in monocultures by target rarity level. A significant divergence of standardized interaction strength from the null demonstrates that biomass in mixtures are significantly different than the expectation in monocultures. **b) Results of linear mixed model examining the effects of categorical interacting rarity level (1–7 and common), continuous phylogenetic distance, and mean adult height on standardized interaction strengths.** Alpha = 0.05.

a) One Sample t-tests				
Response	Target rarity level	DF	t-value	P value
Standardized Interaction Strengths	1	45	3.930	2.895e-04*
	2	23	4.321	2.529e-04*
	3	102	1.702	0.092
	4	63	3.676	4.914e-04*
	5	26	3.673	0.001*
	6	82	2.506	0.014*
	7	42	-2.262	0.029*
	Common	104	-0.665	0.507
b) Analysis of Variance				
Response	Effects	DF	F value	P value
Standardized Interaction Strengths	Interacting Rarity Level	7	3.401	0.001*
	Phylogenetic Distance	1	5.676	0.018*
	Mean Adult Height	1	0.082	0.775
	Interacting Rarity x Phylogenetic Distance	7	1.661	0.116

**Fig 3:**
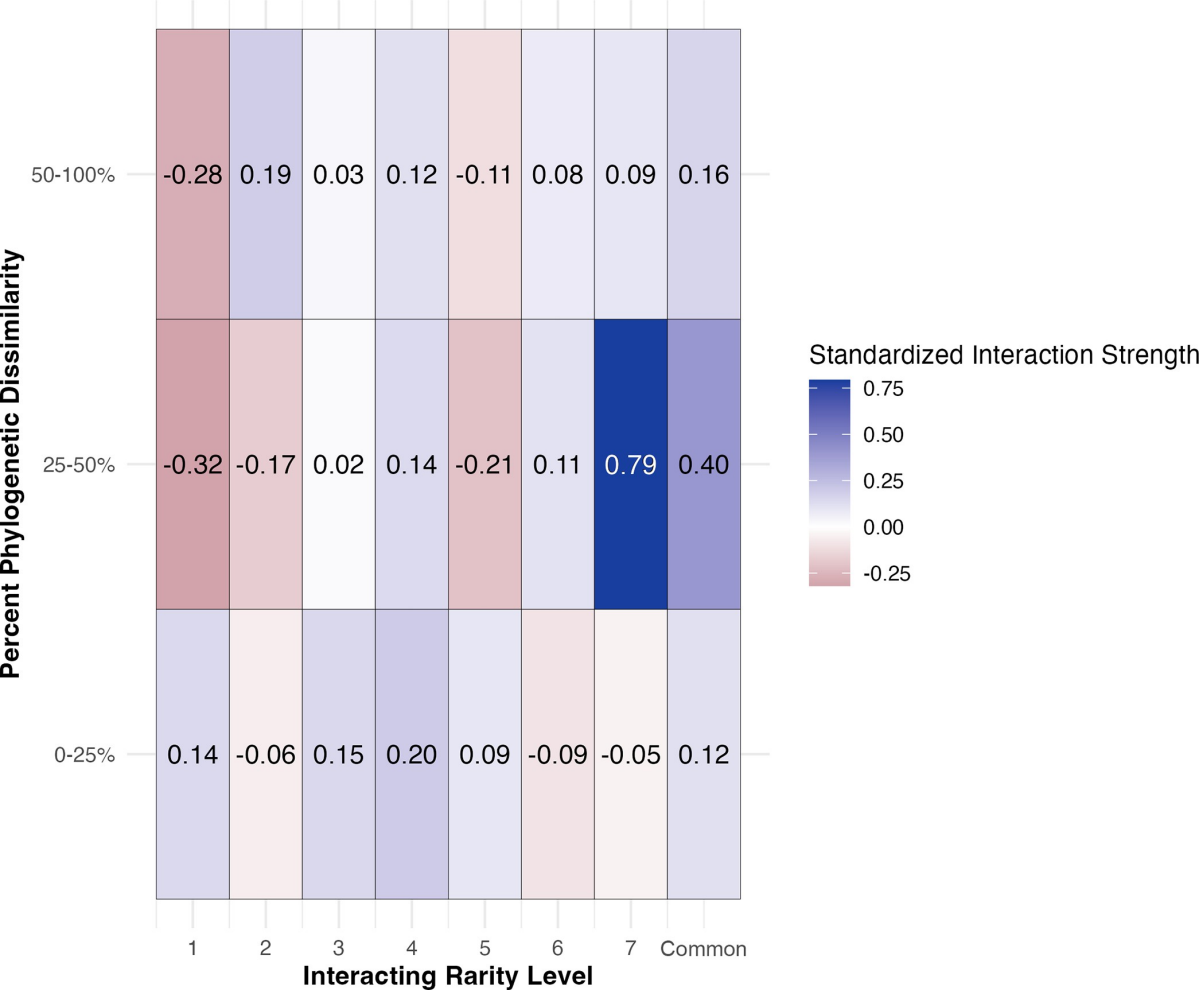
Heat map demonstrating the average standardized interaction strength (of total biomass) of each species pairing across all target rarity levels on the basis of interacting rarity level and percent phylogenetic dissimilarity. Positive interaction strengths representative of synergistic non-additivity in total biomass are represented in shades of blue. Negative interaction strengths representative of antagonistic non-additivity in total biomass are represented in shades of red. Phylogenetic pairings that are 0–25% related are phylogenetically similar, 25–50% related are phylogenetically intermediate, and 50–100% related are phylogenetically dissimilar.

We also found support for hypothesis 2, that the biomass of rare species seedlings will increase when interacting with progressively less rare neighboring species. The interacting rarity level of neighboring species had a significant effect on the total, aboveground, and belowground biomass of all target species across all target rarity levels and phylogenetic pairings (Table 2). Tasmanian eucalypts displayed increased standardized interaction strengths, and therefore synergistic non-additivity in total biomass, when grown with progressively less rare (i.e. level 7) and common species (**Fig 3**). This is most clearly demonstrated in common interacting species, which on average increased the total biomass of all target species, regardless of phylogenetic relatedness, anywhere from 0.12 to 0.4 standard deviations from species-specific monoculture means (Fig 3). All species had the largest increase in total biomass (relative to monocultures) when grown with less rare (i.e. level 7) and common species that are phylogenetically intermediate to the target species (Fig 3). In contrast, all target species had the largest decreases in total biomass when grown with the rarest and phylogenetically intermediate or dissimilar neighboring species (Fig 3).

**Fig 4:**
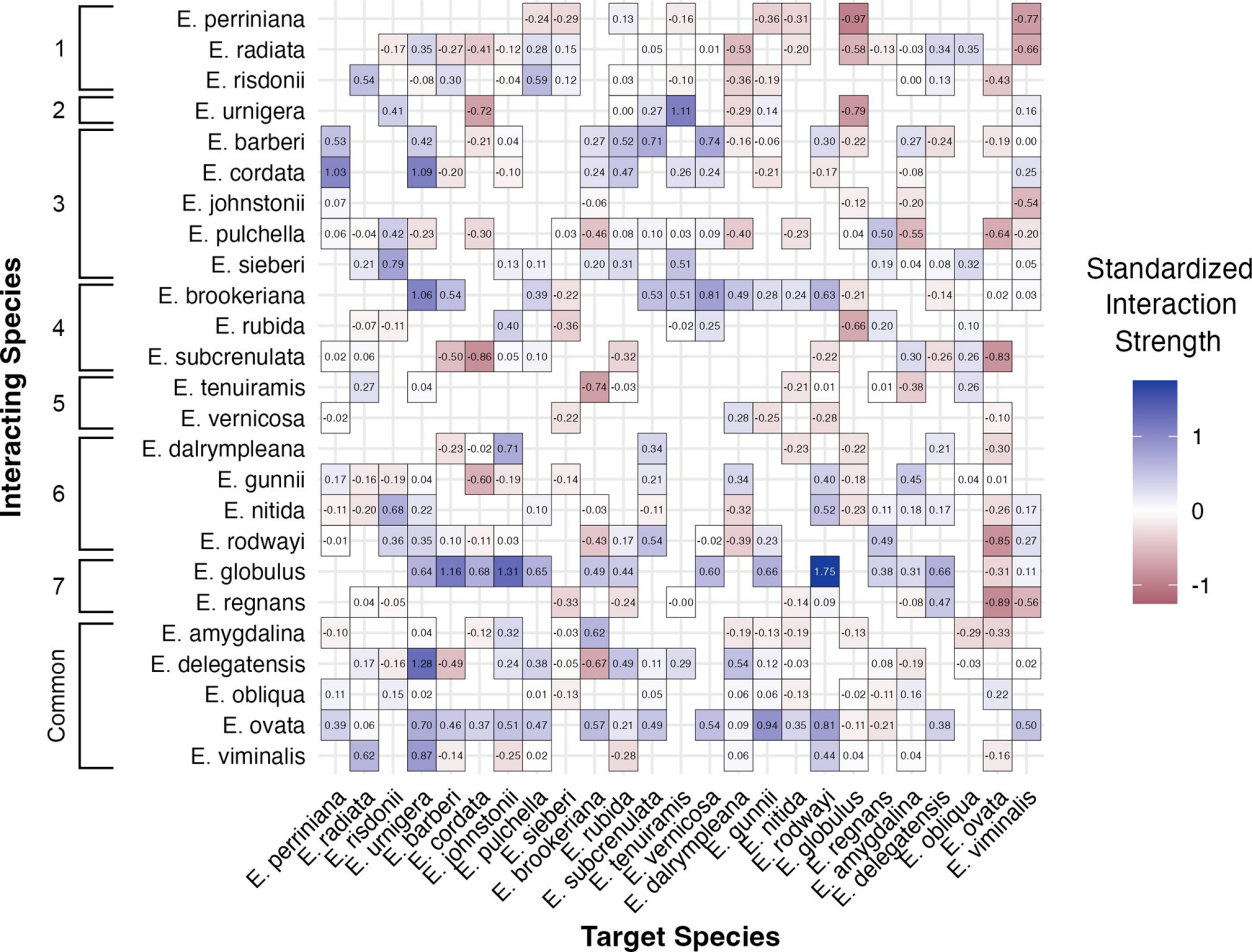
Heat map demonstrating the average standardized interaction strength (of total biomass) of species-specific pairings. Positive standardized interaction strengths represent synergistic non-additivity in total biomass (species-specific pairing outperformed biomass expectation in respective monocultures) and negative standardized interaction strengths represent antagonistic non-additivity in total biomass (species-specific pairing underperformed biomass expectation in respective monocultures). The rarity level of each species is displayed using brackets on the y-axis.

Interestingly, we found species-specific effects on the total biomass of neighboring species, such that certain interacting species either increased or decreased the standardized interaction strength of target species regardless of rarity or phylogenetic distance (**Fig 4 and Table 4**). While more common interacting species tended to increase the total biomass of rare neighbors within phylogenetically intermediate pairings, select species (*E*. *brookeriana*, *E*. *globulus*, and *E*. *ovata*) significantly increased the productivity of all neighboring species (Table 4). Specifically, when planted with *E*. *globulus*, target species on average displayed a 97% increase in total biomass compared to interactions with other species, irrespective of phylogenetic relatedness. This is best seen in *E*. *globulus* x *E*. *barberi* (rarity levels 7 and 3), *E*. *globulus* x *E*. *johnstonii* (rarity levels 7 and 3), and *E*. *globulus* x *E*. *rodwayi* (rarity levels 7 and 6) interactions which demonstrated positive interaction strengths of 1.16, 1.31, and 1.75 respectively. On the other hand, eight species (*E*. *perriana*, *E*. *pulchella*, *E*. *radiata*, *E*. *regnans*, *E*. *rubida*, *E*. *subcrenulata*, *E*. *tenuiramis*, and *E*. *vernicosa*) had significant inhibitory effects on the total biomass of target neighboring species and reduced growth by 31–54% (Table 4).

**Table 4:** Summary average of species-specific effects of neighboring species on target species biomass across all interaction types. Positive target growth values represent increased total biomass of neighboring species, while negative target growth values represent decreased total biomass of neighboring species compared to the mean total biomass of all target species across all mixture types. Alpha = 0.05.

Species	Target Growth (95% CI)	Species	Target Growth (95% CI)
*E*. *amygdalina*	**-** 3% ± 13.8% [-30.2%, 24.2%]	*E*. pulchella*	**-** 31% ± 10.9% [-52.9%, -10%]
*E*. *barberi*	**+** 17% ± 8.9% [-0.05%, 34.6%]	*E*. radiata*	**-** 32% ± 13.5% [-58.9%, -5.8%]
*E*. brookeriana*	**+** 46% ± 7% [32.3%, 59.9%]	*E*. regnans*	**-** 46% ± 12.8% [-70.7%, -20.5%]
*E*. *cordata*	**+** 14% ± 13% [-11.7%, 39.3%]	*E*. *risdonii*	**-** 23% ± 14.6% [-51.4%, 6%]
*E*. *dalrympleana*	**+** 13% ± 10.6% [-7.8%, 33.9%]	*E*. *rodwayi*	**-** 7% ± 11.1% [-28.9%, 14.5%]
*E*. *delegatensis*	**-** 11% ± 9.9% [-30.3%, 8.6%]	*E*. rubida*	**-** 39% ± 13.7% [-65.3%, -11.7%]
*E*. globulus*	**+** 97% ± 7.2% [82.9%, 111.1%]	*E*. *sieberi*	**-** 3% ± 8.2% [-19.3%, 12.7%]
*E*. *gunnii*	**-** 14% ± 18.2% [-49.8%, 21.6%]	*E*. subcrenulata*	**-** 52% ± 14.5% [-80.1%, -23.4%]
*E*. *johnstonii*	**-** 12% ± 26.3% [-63.1%, 39.9%]	*E*. tenuiramis*	**-** 37% ± 11.5% [-59.4%, -14.1%]
*E*. *nitida*	**-** 4% ± 10.8% [-24.9%, 17.3%]	*E*. *urnigera*	**-** 15% ± 14.2% [-43.3%, 12.5%]
*E*. *obliqua*	**-** 19% ± 12.2% [-42.8%, 5.2%]	*E*. vernicosa*	**-** 48% ± 31.1% [-108.7%, 13%]
*E*. ovata*	**+** 49% ± 7.3% [34.3%, 62.9%]	*E*. *viminalis*	**+** 20% ± 10.6% [-0.5%, 41.2%]
*E*. perriniana*	**-** 52% ± 19.4% [-90.2%, -14.3%]		

## Discussion

The composition of rare and common plants in a community is often considered to be the consequence of a combination of stochastic and stabilizing processes which shape the selective pressures imposed upon species according to their frequency and competitive ability. These factors ultimately shape species ranges and dictate where any given combination of species can occur [30]. Overall, our results indicate that rare species can uniquely utilize biotic interactions to increase their biomass, while maintaining lower abundance in communities than their common counterparts. We found support for both of our hypotheses and showed that the growth of rare *Eucalyptus* species is facilitated by intermediately phylogenetically related, and less rare (i.e. level 7) and common neighbors. Rare species biomass increased up to 155% when interacting with phylogenetically similar or intermediate neighbors compared to monocultures. This trend was not observed in common species. All plant species displayed the largest positive non-additive effects on biomass when interacting with phylogenetically intermediate, common species. However, certain species also demonstrated the unique ability to alter the strength and direction of non-additive effects.

Although most studies take an ecological approach to understand the outcomes of biotic interactions involving rare and common species, a relatively small number of studies have demonstrated the critical role of evolutionary history in determining the performance outcomes of species in plant-plant interactions. For example, Kempel et al. [22] found that the degree of phylogenetic relatedness within a community is a strong determinant of competition, such that interactions between phylogenetically similar, rare species are more competitive, while interactions between phylogenetically distant, common species are more facilitative. Our results support Kempel et al. [22] and demonstrate the critical role of phylogenetic relatedness in determining plant productivity in mixture. However, while interactions with rare species had the greatest negative effect on neighboring species’ biomass, phylogenetically similar and intermediate relationships alleviated the disadvantages associated with rare x rare interactions, something not seen in Kempel et al. [22], nor in common eucalypt congeners. Furthermore, differences in species’ performance traits are also affected by biotic interactions, such that smaller, rare species may increase in biomass when interacting with less rare and common species. This facilitative relationship can thus change rare species abundance, competitive ability, and functional role [10,22].

Coexistence theory has also given some insight into how plant-plant interactions may vary with the relatedness of interacting species [31]. Due to the close relationship between a plant’s niche and its evolutionary history, competitive exclusion often occurs in accordance with the phylogenetic relatedness of a plant community [25]. Limiting similarity suggests that competitive exclusion will act to prevent the coexistence of phylogenetically similar species due to the evolutionary conservatism of traits and similar niche spaces. However, our results suggest that the relationship between community composition and phylogenetic relationships varies with species rarity. Specifically, rare species may escape the negative effects of competition under limiting similarity due to frequency-dependent regulation [25]. For instance, rare species at low abundance and frequency within a community may benefit from facilitative interactions with highly productive, common species in high abundance within the same community, therefore enabling the persistence of rare species. Alternatively, the persistent coexistence of rare and common species in communities may be attributed to shared above- and belowground mutualists [32]. Through facilitative interactions with closely and intermediately related species, rare species may engage with and benefit from the specialized systems of resource acquisition and pollination of these neighbors that may go unrecognized and unused by distantly related species. While the mechanisms underlying rare and common species coexistence suggest that evolutionary processes drive patterns of competition and facilitation in plant communities, few studies provide empirical examples of how such evolutionary dynamics affect the strength and direction of plant-plant interactions varying in both relatedness and rarity as we do with Tasmanian *eucalyptus* seedlings. These trends in plant-plant interaction outcomes likely interact with increasing environmental stress and habitat loss to further alter the outcomes and dynamics of plant-plant interactions [33,34].

As climate change is projected to disproportionally affect biodiversity hotspots, leading to heightened environmental instability, rare plants, often found in these regions, may benefit from facilitative, phylogenetically-based interactions that increase their fitness in complex communities [35]. While the phylogenetically based synergistic non-additivity seen in rare species pairings is most likely indicative of facilitation between interacting species, the increased biomass of rare species may instead represent a competitive response indicative of altered resource allocation [36–38]. Additionally, species-specific responses to environmental factors and interacting species can also drive unique trends in the outcomes of plant-plant interactions [39,40]. Regardless, if the productivity of rare plant species is dependent on the rarity level and phylogenetic relatedness of interacting species, as we have demonstrated in Tasmanian *Eucalyptus* seedlings, then traits commonly associated with rarity, are also likely to shift in response to biotic interactions. These shifts in performance traits (productivity, reproduction, and survivability) are expected to have subsequent effects on the competitive potential, functionality, and geographic distribution of rare species, therefore fundamentally altering what it means to be “rare”.

Historically, rare species have been considered inferior competitors when compared to more common species [41–43]. This is often due to a set of traits such as shortened flowering phenology [44], lower seed output [45], smaller reproductive structures [45], and lower biomass [22,41]. Taken together, this combination of traits is often considered innate to the condition of being rare and used to determine conservation priority and status. However, our results suggest that rare species generally increase in biomass when interacting with phylogenetically similar and intermediate, less rare (i.e. level 7) and common species. Moreover, the rarest species demonstrated non-additive effects on biomass in all pairings, regardless of phylogenetic relatedness. Importantly, our results suggest that the traits of rare plant species can be influenced by biotic interactions, in addition to abiotic factors [16], during early seedling growth. One interesting question that arises from these results is: Are rare species inherently rare due to a set of traits or are rare species rare due to the biotic context in which they live? If, as we have demonstrated here, the biomass of rare species is determined in part by biotic factors, as habitat and biodiversity continues to be lost, rare species may benefit from being grown in mixture with more common species. On the other hand, rare species may continue to persist within small geographic ranges and populations, with smaller biomass, due to niche restrictions on community interactions. In any case, the identified increase in total and aboveground biomass of rare species in the proximity of intermediate, more common congeners, could potentially scale up to affect shifts in rare species abundance within communities across rapidly changing landscapes.

## Supporting information

S1 Fig Dendrogram of 25 species of Tasmanian *Eucalyptus*. The phylogeny was constructed using Diversity Array Technology (DArT) markers [20]. Different colored boxes represent four different genetic lineages. The rarity level (1–7) of each species is located next to the species’ name. Rarity levels are ordinally ranked with level 1 representing the rarest species and level 7 representing the least rare species. “C” represents common species.
(DOCX)

S2 Fig Full factorial experimental design consisting of species mixtures varying in rarity and phylogenetic relatedness under varying treatments of Nitrogen (N) fertilization and CO_2_ enrichment. All possible rarity combinations were represented among mixtures varying in phylogenetic distance. Additionally, 25% of mixtures received high N/high CO_2_, 25% of mixtures received high N/low CO_2_, 25% of mixtures received low N/high CO_2_, and 25% of mixtures received low N/low CO_2_.
(DOCX)

S3 Fig Relationship between total seedling biomass and mean adult height of mature Tasmanian *Eucalyptus* species by genetic lineage. Mean adult height represents the average height of mature individuals of 25 species of Tasmanian *Eucalyptus* [46]. Genetic lineages represent distinct genetic groups of eucalypts established using Diversity Array Technology (DArT) markers (S1 Fig).
(DOCX)
