# Supplementary material for: Evolution of rarity and phylogeny determine above- and belowground biomass in plant-plant interactions
Source: PLoS One. 2024 May 20;19(5):e0294839. doi: 10.1371/journal.pone.0294839 (PMC11104619; doi:10.1371/journal.pone.0294839)
Supplement: S1 Fig — The phylogeny was constructed using Diversity Array Technology (DArT) markers [20]. Different colored boxes represent four different genetic lineages. The rarity level (1–7) of each species is located next to the species’ name. Rarity levels are ordinally ranked with level 1 representing the rarest species and level 7 representing the least rare species. “C” represents common species. (DOCX) [file pone.0294839.s001.docx]

**S1 Fig. Dendrogram of 25 species of Tasmanian *Eucalyptus*.** The phylogeny was constructed using Diversity Array Technology (DArT) markers (Wooliver et al. 2017). Different colored boxes represent four different genetic lineages. The rarity level (1-7) of each species is located next to the species’ name. Rarity levels are ordinally ranked with level 1 representing the rarest species and level 7 representing the least rare species. “C” represents common species.

**
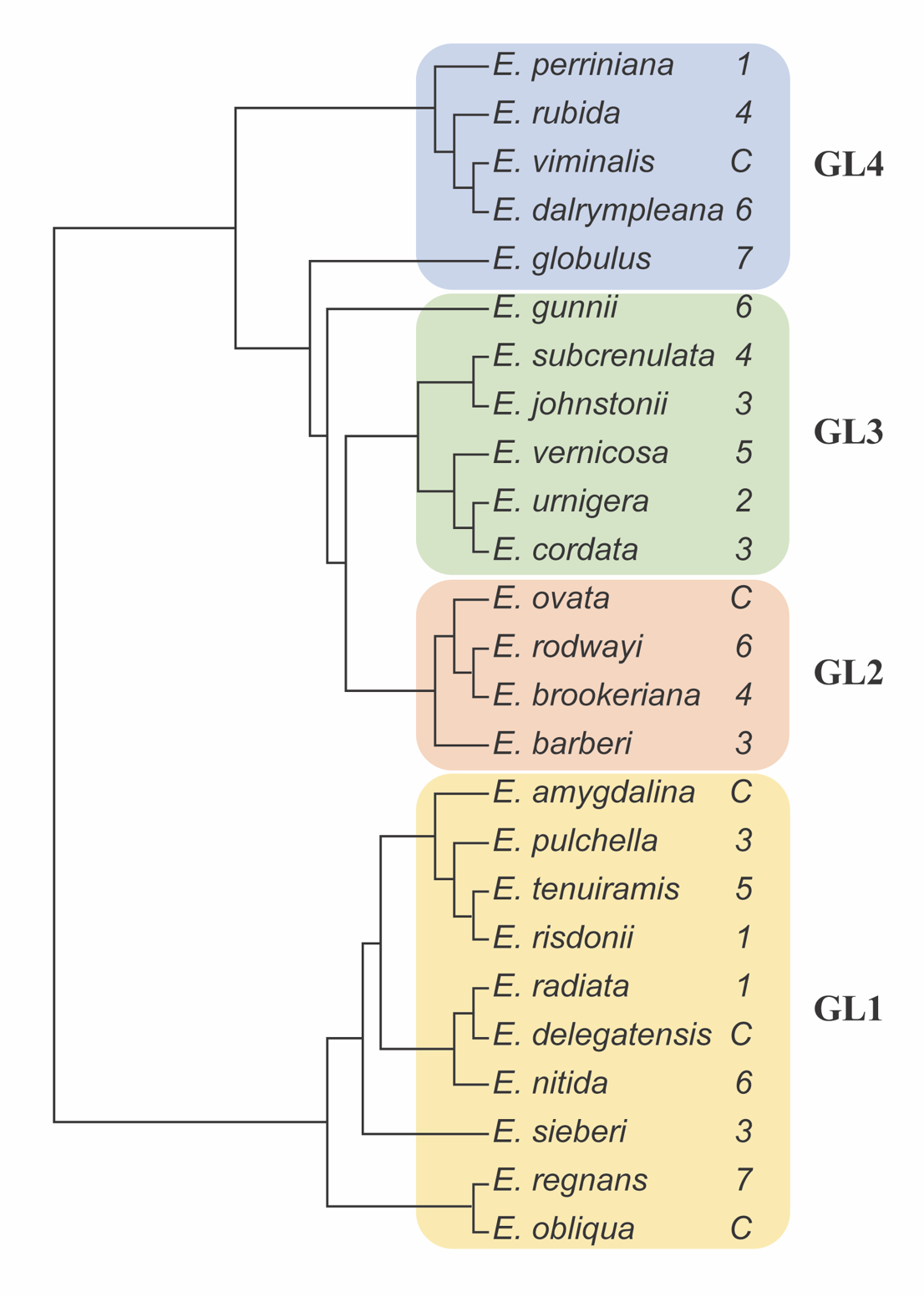
**
