# Supplementary material for: Evolution of rarity and phylogeny determine above- and belowground biomass in plant-plant interactions
Source: PLoS One. 2024 May 20;19(5):e0294839. doi: 10.1371/journal.pone.0294839 (PMC11104619; doi:10.1371/journal.pone.0294839)
Supplement: S2 Fig — All possible rarity combinations were represented among mixtures varying in phylogenetic distance. Additionally, 25% of mixtures received high N/high CO2, 25% of mixtures received high N/low CO2, 25% of mixtures received low N/high CO2, and 25% of mixtures received low N/low CO2. (DOCX) [file pone.0294839.s002.docx]

**S2 Fig. Full factorial experimental design consisting of species mixtures varying in rarity and phylogenetic relatedness under varying treatments of Nitrogen (N) fertilization and CO_2_ enrichment.** All possible rarity combinations were represented among mixtures varying in phylogenetic distance. Additionally, 25% of mixtures received high N/high CO_2_, 25% of mixtures received high N/low CO_2_, 25% of mixtures received low N/high CO_2_, and 25% of mixtures received low N/low CO_2_.

**
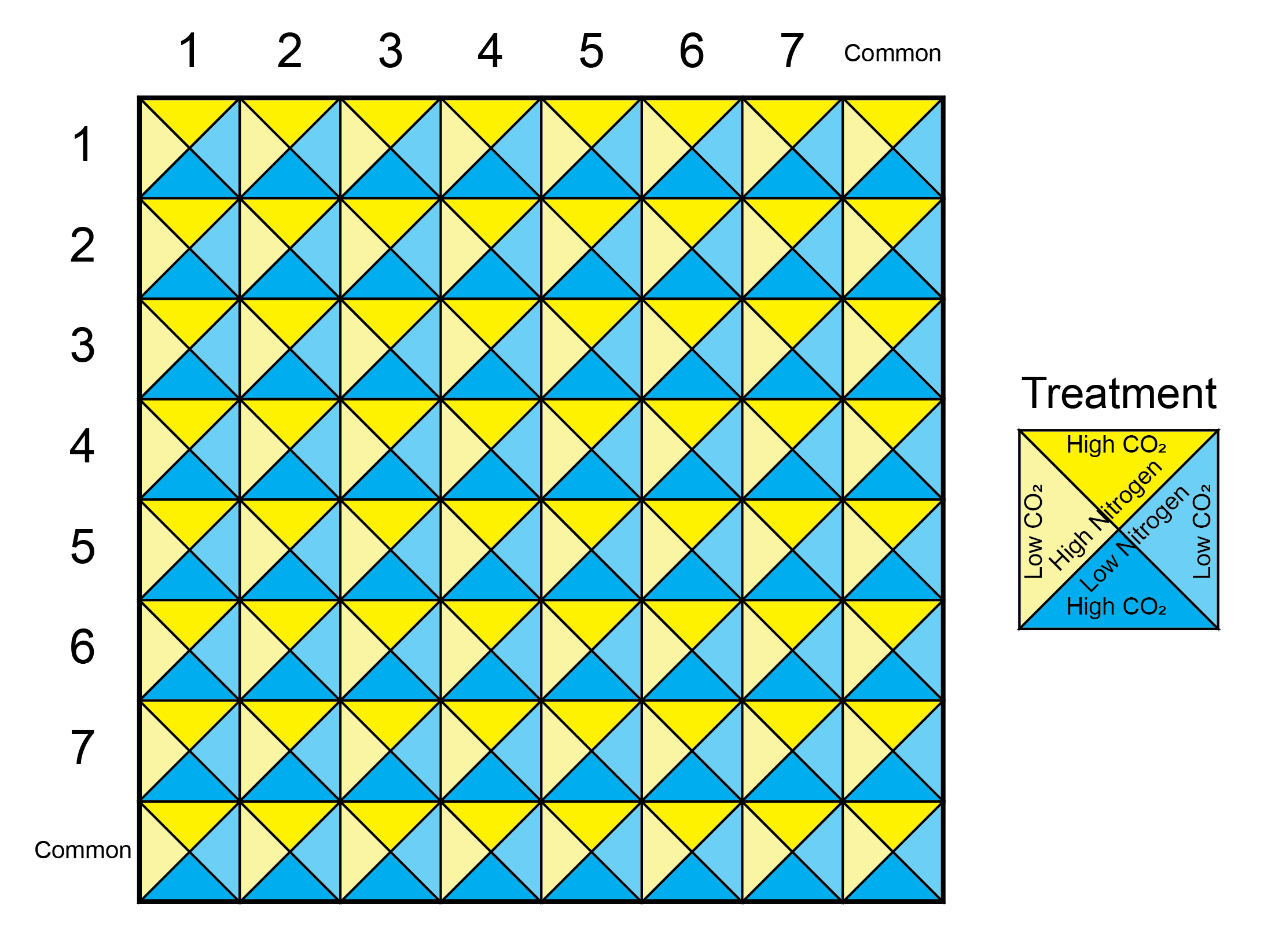
**
