# Supplementary material for: Evolution of rarity and phylogeny determine above- and belowground biomass in plant-plant interactions
Source: PLoS One. 2024 May 20;19(5):e0294839. doi: 10.1371/journal.pone.0294839 (PMC11104619; doi:10.1371/journal.pone.0294839)
Supplement: S3 Fig — Mean adult height represents the average height of mature individuals of 25 species of Tasmanian Eucalyptus [46]. Genetic lineages represent distinct genetic groups of eucalypts established using Diversity Array Technology (DArT) markers (S1 Fig). (DOCX) [file pone.0294839.s003.docx]

**S3 Fig. Relationship between total seedling biomass and mean adult height of mature Tasmanian *Eucalyptus*** **species** **by genetic lineage.** Mean adult height represents the average height of mature individuals of 25 species of Tasmanian *Eucalyptus* [46]. Genetic lineages represent distinct genetic groups of eucalypts established using Diversity Array Technology (DArT) markers (S1 Fig).

**
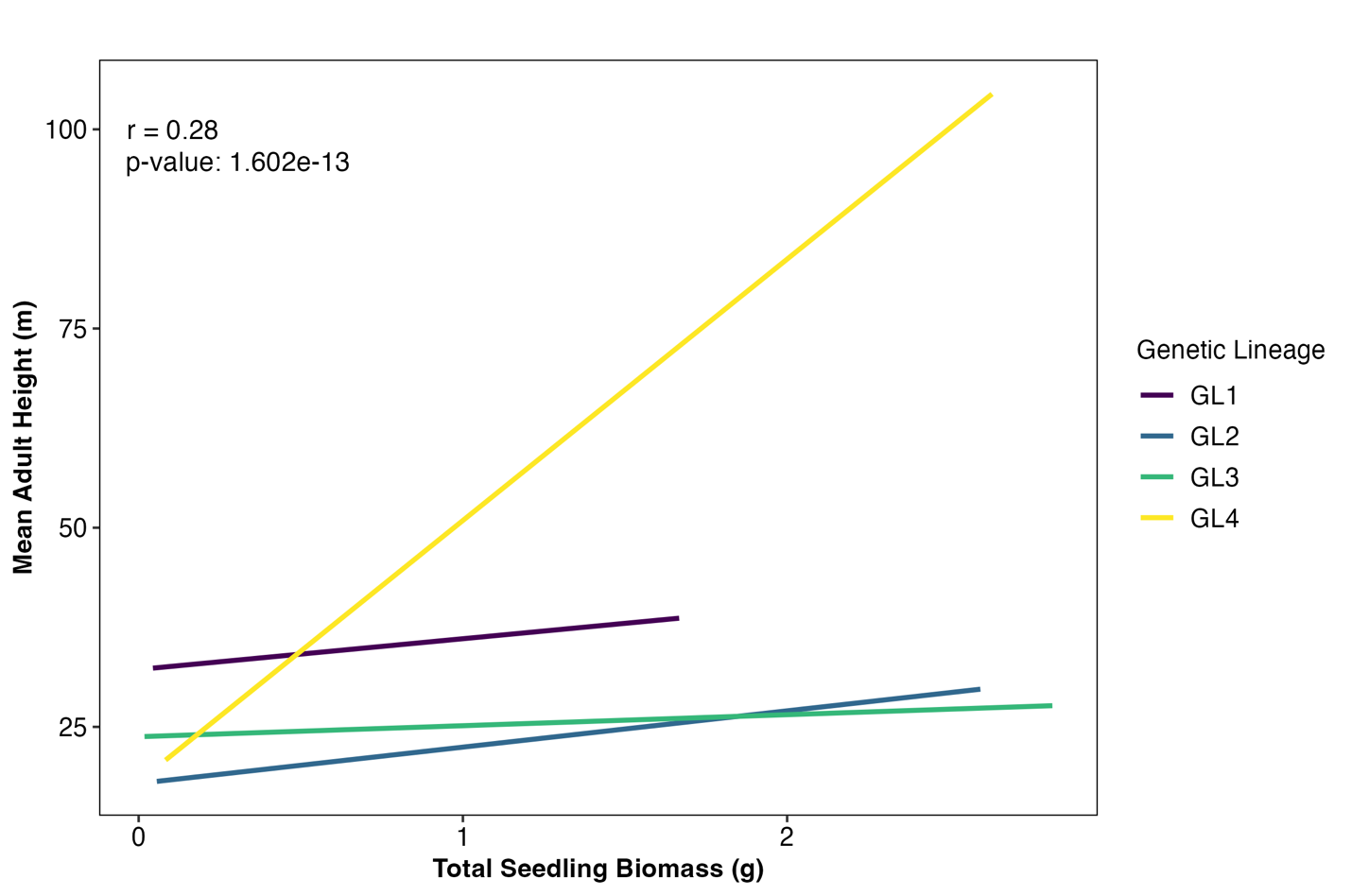
**
